# Evaluation of the EndoPAT as a Tool to Assess Endothelial Function

**DOI:** 10.1155/2012/904141

**Published:** 2012-02-14

**Authors:** M. Moerland, A. J. Kales, L. Schrier, M. G. J. van Dongen, D. Bradnock, J. Burggraaf

**Affiliations:** Centre for Human Drug Research, Department of Vascular Medicine, Zernikefdreef 10, 2333 CL Leiden, The Netherlands

## Abstract

Endothelial dysfunction is a potential target for (pharmaceutical) intervention of several systemic pathological conditions. We investigated the feasibility of the EndoPAT to evaluate acute changes in endothelial function with repeated noninvasive measurements and assessed its discriminating power in different populations. Endothelial function was stable over a longer period of time in renally impaired patients (coefficient of variation 13%). Endothelial function in renally impaired and type 2 diabetic patients was not decreased compared to healthy volunteers (2.9 ± 1.4 and 1.8 ± 0.3, resp., versus 1.8 ± 0.5, *P* > 0.05). The EndoPAT did not detect an effect of robust interventions on endothelial function in healthy volunteers (glucose load: change from baseline 0.08 ± 0.50, 95% confidence interval −0.44 to 0.60; smoking: change from baseline 0.49 ± 0.92, 95% confidence interval −0.47 to 1.46). This suggests that at present the EndoPAT might not be suitable to assess (changes in) endothelial function in early-phase clinical pharmacology studies. Endothelial function as measured by the EndoPAT could be physiologically different from endothelial function as measured by conventional techniques. This should be investigated carefully before the EndoPAT can be considered a useful tool in drug development or clinical practice.

## 1. Introduction

Endothelial dysfunction is an early predictor of cardiovascular disease [1–3], and might be the causal pathological mechanism of a variety of metabolic diseases, also referred to as the common soil hypothesis [4]. Endothelial function has been shown to be impaired in patients with coronary artery disease, type II diabetes mellitus, hypertension, obesity, renal failure, and hypercholesterolemia [5–9]. It is conceivable that improvement of endothelial function will be an important target in the treatment of these conditions. Therefore, availability of methodology that can be used to reliably assess the effects of (pharmacological) treatments on endothelial function is of critical importance.

Endothelial dysfunction is commonly described as the inability of the artery to sufficiently dilate in response to an appropriate endothelial stimulus. It can be assessed by measurement of the arterial pulse wave at a finger artery or by the measurement of flow-mediated dilation (FMD) of the brachial artery after occlusion of the blood flow. Although the exact mechanisms causing FMD are not entirely known, the main mechanism inducing FMD is thought to be an increase in shear stress, leading to the release of nitric oxide from endothelial cells which causes blood vessel dilation [10]. Currently, FMD is assessed clinically in a noninvasive manner using high-resolution ultrasound of the brachial artery. The technique is widely used and has been shown to be a suitable tool to assess endothelial dysfunction. However, the method has several disadvantages: it is operator dependent [11], and as FMD is measured at one arm only, there are no possibilities to correct for potential measurement-induced changes in the systemic hemodynamics, such as those resulting from alterations in the autonomous nervous system tone. To overcome these problems, the EndoPAT was developed. This device allows non-invasive measurement of vasoreactivity without the disadvantages of conventional ultrasound measurement. The EndoPAT detects plethysmographic pressure changes in the finger tips caused by the arterial pulse and translates this to a peripheral arterial tone (PAT). Endothelium-mediated changes in vascular tone after occlusion of the brachial artery are reflecting a downstream hyperemic response, which is a measure for arterial endothelial function [12]. Measurements on the contralateral arm are used to control for concurrent nonendothelium-dependent changes in vascular tone. In addition, the EndoPAT provides a measure for arterial stiffness: the augmentation index (AI). In theory, the EndoPAT could be a useful device in clinical research as the test is easy to perform, not operator-dependent, and with comprehensive automatic analysis. In a group of 89 adult patients suffering from chest pain, peripheral arterial tone correlated positively with FMD [12]. In the Framingham study, a significant inverse relation was observed between endothelial function as determined by the EndoPAT (“EndoScore” or reactive hyperemia index, RHI) and multiple cardiovascular risk factors (male sex, body mass index, total/HDL cholesterol, diabetes, smoking, and lipid-lowering treatment) [13]. The EndoScore was reported to be significantly decreased in patients with coronary artery disease, hypertension, hyperlipidemia, diabetes, glucose intolerance, and tobacco users (group sizes of 15 to 70 subjects) [12, 14–18]. Several EndoPAT studies have demonstrated an improvement in endothelial function as a result of lifestyle modification (smoking cessation, and dietary change) [19–22] or prolonged pharmacological intervention [23, 24]. However, there is only limited information on the performance of the EndoPAT for repeated measurements in a relatively short time frame. This information is pertinent as in many clinical (pharmacology) studies repeated measures are performed in populations consisting of 6 to 12 subjects.

Therefore, we performed a series of experiments to investigate the feasibility of the EndoPAT to evaluate acute changes in endothelial function and arterial stiffness with repeated measurements and to assess the discriminating power of the EndoPAT in different populations. First, we investigated the variability of endothelial function and arterial stiffness, as measured by the EndoPAT, in patients with chronic kidney failure on three different days. Endothelial function, as assessed by high-resolution ultrasound, is known to be severely impaired in this patient population, despite intensive treatment (30–60% reduction) [25–28]. In addition, we measured endothelial function and arterial stiffness in patients with diabetes mellitus type 2, another patient population with a strongly reduced endothelial reactivity as determined using conventional techniques (30–35% reduction) [29–31]. Finally, we investigated the capability of the device to detect changes in endothelial function induced by two acute and robust interventions (smoking and glucose administration) in healthy volunteers. Cigarette smoking acutely impairs endothelial function in healthy volunteers by causing oxidative stress and reducing the production of nitric oxide due to the free radicals present in cigarette smoke [2, 32]. Also high blood glucose levels lead to an attenuated endothelial function, as has been demonstrated by plethysmography and high-resolution ultrasound [33, 34].

## 2. Materials and Methods

The experiments were approved by the Medical Ethics Committee of Leiden University Medical Center (LUMC). Endothelial function and arterial stiffness were assessed in 6 renal patients (creatinine clearance between 30 and 70 mL/min) and 16 patients with diabetes mellitus type 2 (8 using metformin and 8 using metformin plus sulfonylurea). Intervention studies were performed in apparently healthy males and females, not using any medication. In all experiments, subjects were fasted for at least 3 hours before EndoPAT measurements.

### 2.1. Protocol

Reactive hyperemia index (RHI), which is a measure for endothelial function, and augmentation index (AI), which is a measure for arterial stiffness, were assessed using the EndoPAT 2000 device (Itamar Medical, Israel). Both measures were calculated using a computerised automated algorithm (software version 3.1.2) provided with the device. Measurements were performed according to the manufacturer's instructions. Briefly, the subjects were in supine position for a minimum of 20 minutes before measurements, in a quiet, temperature-controlled (21–24°C) room with dimmed lights. The subjects were asked to remain as still as possible and silent during the entire measurement period. Each recording consisted of 5 minutes of baseline measurement, 5 minutes of occlusion measurement, and 5 minutes postocclusion measurement (hyperemic period). Occlusion of the brachial artery was performed on the nondominant upper arm. The occlusion pressure was at least 60 mmHg above the systolic blood pressure (minimally 200 mmHg, and maximally 300 mmHg).

### 2.2. Renal Patient Study Part

Endothelial function and arterial stiffness were investigated in six patients with chronic kidney failure (subject characteristics in Table 1, glomerular filtration rate was estimated by calculation of creatinine clearance using a 24-hour urine collection). All subjects were on maintenance therapy for treatment of hypertension. All measurements were performed in the morning. Per subject, 3 measurements were performed, separated by one to two weeks. Patients were allowed to use medication needed for their medical condition, except for corticosteroids and erythropoietic medication within one month before study participation.

### 2.3. Diabetic Patient Study Part

Endothelial function and arterial stiffness were investigated in 16 patients with diabetes mellitus type 2 (subject characteristics in Table 2). All subjects were on oral antidiabetics to control glucose metabolism: 8 subjects were using metformin, and 8 subjects were using metformin plus sulfonylurea. Per subject, two EndoPAT measurements were performed: one time during continuation of the antidiabetic therapy and one time after two weeks of therapy discontinuation, with the sequence randomized. All measurements were performed in the evening. Patients were allowed to use medication needed for their medical condition, except for medication known to affect glucose homeostasis (other than biguanides and sulphonylurea), anti-inflammatory drugs, nonselective beta blockers, oral anticoagulants, and systemic glucocorticoids or other immunosuppressive drugs.

### 2.4. Oral Glucose Intervention in Healthy Volunteers

Six nonsmoking female subjects (mean age  23 ± 3  years) participated in the oral glucose intervention study part. After the baseline EndoPAT measurement was performed, an oral glucose solution (75 g glucose in 300 mL) was consumed within 4 minutes. At 30 minutes and 90 minutes after the oral glucose consumption, EndoPAT measurements were performed.

### 2.5. Smoking Intervention in Healthy Volunteers

Six male cigarette smoking subjects (mean age  32 ± 10  years) participated in this study part. The number of cigarettes per subject per day ranged between 1 and 20 cigarettes. Subjects refrained from smoking until at least 2 hours before the start of the study. After a baseline measurement was performed, the participants smoked a cigarette (tar 10 mg, nicotine 0.8 mg and carbon monoxide 10 mg) within 4 minutes. At 30 minutes and 90 minutes after smoking, the measurements were repeated.

### 2.6. Data Analysis and Statistics

Unpaired *t*-tests were used to compare RHI and AI between healthy volunteers and patients and between diabetic patients using metformin only and patients using metformin plus sulfonylurea, while paired *t*-tests were used to compare RHI and AI between diabetic patients who continued their medication and patients who discontinued their medication. The intervention experiments were carried out in groups of 6 healthy volunteers; a sample size based on the observed intraindividual variability in RHI and the level of impairment of endothelial function determined by conventional techniques (high-resolution ultrasound, plethysmography) in intervention studies as reported in literature. Our experiments were powered such that a group size of 6 would have 80% power to detect a 25% change in baseline RHI using a 2-sided alpha of 5%. Paired *t*-tests were used to compare the difference in RHI between baseline and the postintervention measurements, which was considered to be statistically significant when *P* value < 0.05.

## 3. Results

To assess the performance of the EndoPAT, we investigated the variability of endothelial function (RHI) and arterial stiffness (AI) in patients with chronic kidney failure on three different days, separated by one to two weeks. Average RHI over three days was 2.9 ± 1.4 (Table 3). For most patients, the RHI values exceeded a value of 2 (range: from 1.7 to 5.5). Official reference values for RHI are not available, but in general RHI values below 2 are categorized as endothelial dysfunction, whereas higher RHI values are considered normal or improved endothelial function (Itamar product information). The average intra-individual variability for the RHI was 13% (ranging from 1% to 29% for individuals, data not shown). The general estimate for interindividual variability in RHI was 50% (Table 3). Average AI over three days was 26.1 ± 13.9 (Table 4). This is in line with the expectation, as normal arterial stiffness is defined by an AI between −30% and −10%, increased arterial stiffness by an AI between −10% and 10%, and abnormal arterial stiffness by an AI above 10%. The mean intra-individual coefficient of variation was 37% (ranging from 13% to 67%, data not shown). These data indicate that whereas endothelial function is a relatively stable measure over a longer period of time, arterial stiffness as determined by the EndoPAT is rather variable.

Next, we measured endothelial function and arterial stiffness in patients with diabetes mellitus type 2, 8 subjects using metformin and 8 subjects using metformin plus sulfonylurea. Endothelial function and arterial stiffness were determined during continuation of the antidiabetic therapy and after two weeks of therapy discontinuation. Arterial stiffness, as determined by AI, was not different between patients using metformin or metformin plus sulfonylurea or between patients continuing and discontinuing antidiabetic medication (Table 4: 5.9 ± 9.4 versus 9.0 ± 13.0 and 14.3 ± 18.8 versus 16.3 ± 16.0, resp.). However, RHI was significantly higher in patients using a combination of metformin plus sulfonylurea, compared to patients using metformin only (Table 3: 2.5 ± 0.7 versus 1.8 ± 0.3 in patients continuing therapy and 2.7 ± 1.1 versus 1.8 ± 0.4 in patients discontinuing therapy; *P* = 0.02 and 0.04 resp.). The increasing effect of sulfonylurea treatment on RHI was independent on continuation or discontinuation of the use of antidiabetics.

**Figure 1:**
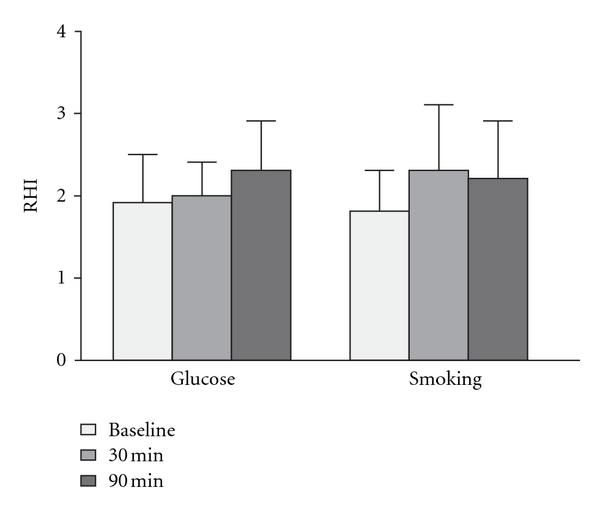
Interventions and endothelial function. Reactive hyperemia index was assessed before and at 30 and 90 minutes after an oral glucose load (left) or after cigarette smoking (right).

Finally, we investigated the capability of the EndoPAT to detect changes in endothelial function induced by two acute interventions in healthy volunteers. Neither the oral glucose load nor the smoking intervention resulted in significant effects on endothelial function (Figure 1). For the glucose intervention, the difference in RHI from baseline measurement was 0.08 ± 0.50 (95% CI, confidence interval: from −0.44 to 0.60) for the 30 min assessment and 0.44 ± 0.86 (95% CI: from −0.46 to 1.34) for the 90 min assessment. For the smoking intervention, the difference in RHI from baseline measurement was 0.49 ± 0.92 (95% CI: from −0.47 to 1.46) for the 30 min assessment and 0.39 ± 0.53 (95% CI: from −0.16 to 0.95) for the 90 min assessment.

AI and RHI were compared between the investigated patient groups and the healthy volunteer group. As expected, AI was higher in renal patients compared to healthy subjects (Table 4: 26.1 ± 13.9 in patients versus −6.0 ± 14.2 in healthy volunteers, *P* = 0.001). AI was also higher in diabetic patients compared to healthy subjects, independent of type of oral antidiabetics (metformin or metformin plus sulfonylurea) or continuation or discontinuation of therapy (Table 4: 5.9 ± 9.4, 9.0 ± 13.0, 14.3 ± 18.8, and 16.3 ± 16.0, resp., in patients versus −6.0 ± 14.2 in healthy volunteers, 0.01 < *P* < 0.05). However, there was no difference in RHI between the renal patients and the healthy volunteers (Table 3: 2.9 ± 1.4 in patients versus 1.8 ± 0.5 in healthy volunteers, *P* = 0.15). Furthermore, RHI values did not differ between diabetic patients using metformin and healthy volunteers (Table 3: 1.8 ± 0.3 in patients continuing therapy and 1.8 ± 0.4 in patients discontinuing therapy versus 1.8 ± 0.5 in healthy volunteers). RHI was significantly higher in diabetic patients using a combination of metformin plus sulfonylurea compared to healthy volunteers (Table 3: 2.5 ± 0.7 in patients continuing therapy and 2.7 ± 1.1 in patients discontinuing therapy; *P* = 0.04 and 0.01 resp. versus healthy volunteers).

## 4. Discussion

Endothelial dysfunction is present in several systemic pathological conditions [5–9], associated with considerable morbidity and mortality. As a consequence, endothelial dysfunction is expected to gain interest as potential target for (pharmaceutical) intervention within the coming years. Currently, endothelial function is assessed mainly by high-resolution ultrasound of the brachial artery. However, this technique has important practical limitations: it is strongly operator-dependent [11], and it offers no correction for autonomous activation of the nervous system, as vascular dilation is only studied in one arm. To overcome these problems, the EndoPAT was developed, allowing noninvasive measurement of endothelial function via assessment of reactive hyperemia. In addition, the EndoPAT provides a measure for arterial stiffness. Although in several studies the EndoPAT appeared to be feasible to demonstrate improvement of endothelial function as a result of lifestyle modification or pharmacological intervention [19–24], the information in literature on the performance of the EndoPAT in intervention trials is limited. Therefore, we performed a series of experiments to investigate the feasibility of the EndoPAT to evaluate acute changes in endothelial function with repeated measurements and to assess the discriminating power of the EndoPAT for endothelial function and arterial stiffness in different populations.

We assessed the variability of endothelial function (by RHI) and arterial stiffness (by AI), as measured by the EndoPAT, in patients with impaired renal function on three different days. Next, we measured endothelial function and arterial stiffness in patients with diabetes mellitus type 2. Finally, we investigated the applicability and feasibility of the EndoPAT to detect changes in endothelial function in healthy volunteers after interventions known to be associated with robust acute changes in endothelial function in an adequately powered experiment.

Generally, augmentation index, calculated from carotid, aortic, or radial artery pressure waves using conventional techniques, is a reliable and reproducible measure to define arterial stiffness [35]. However, the influence of variables such as heart rate and vasomotor tone of the arterial system can affect the variability of the technique [36]. We demonstrated that when using the EndoPAT, the intra-individual variability in AI was substantial over a longer period of time (CV: coefficient of variation, 37%). This indicates that arterial stiffness as determined by the EndoPAT is also not a stable measure, which limits its usefulness to assess the effects of (pharmacological) interventions. Arterial stiffness as measured by the EndoPAT was higher in renally impaired patients with vascular disease compared to healthy subjects. This is in line with reports in literature in patient populations: compared to healthy volunteers, arterial stiffness is increased in a variety of pathological conditions, such as coronary artery disease, metabolic syndrome, and chronic kidney disease [37–39].

Compared with arterial stiffness, RHI proved to be a more stable measure (CV 13%). Surprisingly, endothelial function, as determined by reactive hyperemia index, was not impaired in the renal patient group. Importantly, observed RHIs in the patient population were scattered over a broad range, covering both endothelial dysfunction (RHI < 2) and exceptionally good endothelial function (RHI > 3). Given the fact that all subjects were renally impaired and treated for hypertension, it is very unlikely that 4 out of 6 patients had an (exceptionally) good endothelial function. Obviously, all patients used medication with pertinent effects on endothelial function (Table 1: statins, ACE inhibitors, and calcium channel blockers) [40], but literature data suggest that despite optimal (cardiovascular) therapy, a considerable increased risk for cardiovascular morbidity in this population still exists [41]. Importantly, although most chronic kidney disease patients use antihypertensive medication and other drugs that could affect endothelial function (statins, potassium, and ACE inhibitors), they still exhibit a significantly impaired endothelial function as demonstrated by laser Doppler flowmetry (32% reduction) [25]. Interestingly, Gordon et al. reported that pulse contour analysis obtained by finger plethysmography, which is comparable with the EndoPAT methodology, may not be suitable to measure endothelial function in subjects with extensive coronary artery disease, as no effect of administration of strong vasodilators (salbutamol and nitroglycerin) could be observed [42].

We investigated whether the EndoPAT could discriminate in vascular function between healthy volunteers and patients with diabetes mellitus type 2. As expected, arterial stiffness was higher in diabetic patients compared to healthy subjects, irrespective of treatment with oral antidiabetics. However, endothelial function (as measured by the EndoPAT) was not impaired in diabetic patients using metformin compared to healthy volunteers, neither during continuation of treatment nor after two weeks of treatment discontinuation. This is contrasting with literature, which demonstrates that endothelial function, measured using conventional techniques (endothelium-dependent flow-mediated dilatation of the brachial artery, by ultrasound), is substantially impaired in diabetic patients (reduction of endothelial function versus healthy controls or subjects with coronary artery disease without diabetes ranging from 27% to 43%) [29–31]. This discrepancy could not be explained by an effect of treatment with oral antidiabetics, as RHI was assessed both during treatment continuation and after a two-week period of treatment discontinuation.

RHI was significantly increased in patients using a combination of metformin plus sulfonylurea, compared to healthy volunteers and to patients using metformin only. This stimulating effect of sulfonylurea treatment on RHI was still observed after two weeks of treatment discontinuation, implicating a long-lasting effect of sulfonylurea on endothelial function. This is remarkable as the general point of view is that the use of pancreatic *β*-cell-specific sulfonylurea (i.e., glimepiride, as used by 6 out of 8 diabetic subjects in our study) does not affect endothelial function [43–45]. Possibly, endothelial function as measured by the EndoPAT (RHI) is a physiologically different process than endothelial function as measured by conventional techniques such as high-resolution ultrasound (endothelium-dependent FMD). The exact nature of this difference is currently unknown, and should be investigated in detail before the EndoPAT can be considered a useful tool in drug development or clinical practice.

We evaluated the performance of the EndoPAT to detect the effects of two different acute interventions on RHI in healthy subjects. The literature shows that FMD decreases by 40–65% after smoking one cigarette, which is detected directly after smoking and lasts for 1 hour [2, 32, 46, 47]. Also hyperglycaemia, induced by the administration of a standardised oral glucose load, has been reported to acutely lead to a 25–45% impairment of endothelial function, as assessed by FMD or forearm blood flow plethysmography [33, 34, 48]. However, we were unable to replicate these findings on endothelial function using the EndoPAT, demonstrating that the device is currently not suitable to detect acute changes in endothelial function after different robust interventions. There could be several explanations for our findings. In the automated analysis, the EndoPAT software uses a fixed time frame during the hyperemic response to calculate the RHI. Inspection of the data and manual analysis of the RHI showed that the maximal hyperemic response does not always occur in the same time period after occlusion (data not shown), an observation that is supported by literature. For example, the time course of FMD is strongly influenced by age: comparison of young and older subjects indicates that the time frame to reach maximal vasodilatation after occlusion is significantly prolonged in older subjects [49]. This may be remedied by refinement of the EndoPAT software to allow for interindividual differences in hyperemic time course. Although inter-individual differences in hyperemic time course is a potentially confounding factor when using the automatic analysis, manual analysis of our data did not result in significantly different findings (data not shown). There are some indications that endothelial function is subject to a circadian rhythm, with a lower reactive hyperemic response in the morning [50], but this is not unambiguously supported [51–54]. In fact, the presumed circadian variability is probably more related to changes in physical activity, blood pressure and shear stress, and changes in plasma lipids. Whatever the explanation may be, our experiments were performed within a short fixed time frame of maximally 2 hours, in a fasted condition and in complete rest, thereby reducing the influence of these confounding factors. As a consequence, it is unlikely that circadian variability has influenced our measurement outcomes. Finally, it is possible that previous experiments using ultrasound or plethysmography to assess the acute effect of an intervention on endothelial function are flawed because measurements were performed (in the majority of the cases) in the vasculature of one arm only, and thus relatively uncontrolled for concomitant systemic hemodynamic changes. We consider this a theoretical explanation, but unlikely. We are well aware that the group sizes of our intervention experiments were small and that no formal control groups were included. However, the experiments were sufficiently powered to detect intervention-induced changes in endothelial function at effect sizes that are reported in literature using FMD or forearm blood flow plethysmography.

In conclusion, whereas the reactive hyperemia index (a measure for endothelial function), as determined by the EndoPAT, is rather stable over time, the augmentation index (a measure for arterial stiffness) showed substantial intra-individual variability, limiting its value for evaluation of (pharmacological) interventions. Surprisingly, the EndoPAT did not demonstrate differences in endothelial function between healthy volunteers and renally impaired patients with known vascular disease or diabetic patients. In the latter patient group, an unexplained improving effect of sulfonylurea on reactive hyperemia index was demonstrated by the EndoPAT. This could indicate that endothelial function as measured using the EndoPAT might be physiologically different from endothelial function as measured by conventional techniques. Furthermore, the EndoPAT was not useful to detect the effect of robust interventions on endothelial function while the experiments were adequately powered. Taken together, our findings suggest that the EndoPAT is at present not suitable to assess (changes in) endothelial function and arterial stiffness in populations with sizes that are commonly employed in clinical pharmacology studies.

## Figures and Tables

**Table 1:** Renal patients, baseline characteristics.

Subject	Gender	Age (years)	BMI (kg/m^2^)	GFR (mL/min)	BP (mmHg)	HR (bpm)	Maintenance therapy
1	M	59	24	45	140/83	63	a, b, c
2	M	63	27	47	134/82	54	a, c, d, e, f, g
3	F	57	20	41	138/61	67	a, c, e, h, i
4	F	46	19	51	120/73	58	b, h
5	F	59	22	55	150/86	64	a, c
6	M	65	32	69	140/75	75	a, b, j

^a^statin, ^b^calcium channel blocker, ^c^ACE inhibitor, ^d^thiazide diuretic, ^e^platelet aggregation inhibitor, ^f^angiotensin II receptor antagonist, ^g^intestinal potassium binder, ^h^potassium chloride, ^i^acetylsalicylic acid, and ^j^ *β*1 receptor blocker.

BP: blood pressure (supine).

BMI: body mass index.

GFR: glomerular filtration rate.

HR: heart rate (supine).

**Table 2:** Diabetic patients, baseline characteristics.

	Metformin group	Metformin plus sulfonylurea group
Number (male/female)	8 (7/1)	8 (8/0)
Age (years)	56 ± 8	58 ± 6
BMI (kg/m^2^)	27 ± 3	27 ± 4
Systolic BP (mmHg)	137 ± 7	142 ± 7
Diastolic BP (mmHg)	83 ± 6	79 ± 8
HR (bpm)	70 ± 9	74 ± 11
Glucose (mM)	6.3 ± 0.8	6.8 ± 3.2
HbA_1c_ (mM)	6.4 ± 0.5	6.1 ± 0.6

(Mean ± SD).

BP: blood pressure (supine).

BMI: body mass index.

HR: heart rate (supine).

**Table 3:** Reactive hyperaemia index.

Population	*N*	Therapy	Study period	mean ± SD	min–max	CV
Renal patients	6	N/A	N/A^#^	2.9 ± 1.4	1.7–5.5	50%
Diabetic patients	8	Metformin	Cont.	1.8 ± 0.3	1.5–2.2	16%
Diabetic patients	8	Metformin	Discont.	1.8 ± 0.4	1.1–2.4	25%
Diabetic patients	8	Metformin + SA	Cont.	2.5 ± 0.7^∗,∗∗^	1.7–3.7	28%
Diabetic patients	8	Metformin + SA	Discount.	2.7 ± 1.1^∗,∗∗^	1.7–4.9	41%
Healthy volunteers	12	N/A	N/A	1.8 ± 0.5	1.3–2.8	29%

SA: sulfonylurea; SD: standard deviation; CV: coefficient of variation.

Cont: continuation of therapy; discount: 2 weeks of therapy discontinuation.

^#^visit 1, 2, and 3 and averaged (see materials and methods).

*P < 0.05 versus healthy volunteers.

**P < 0.05 versus diabetic patients on metformin only.

**Table 4:** Augmentation index.

Population	*N*	Therapy	Study period	mean ± SD	min–max
Renal patients	6	N/A	N/A^#^	26.1 ± 13.9*	13.3–52.0
Diabetic patients	8	Metformin	cont	5.9 ± 9.4**	−8.0–22.0
Diabetic patients	8	Metformin	discont	14.3 ± 18.9**	1.0–55.0
Diabetic patients	8	Metformin + SA	cont	9.0 ± 13.0^∗∗, ∗∗∗^	−15.0–24.0
Diabetic patients	8	Metformin + SA	discont	16.3 ± 16.0^∗∗, ∗∗∗^	−6.0–32.0
Healthy volunteers	12	N/A	N/A	−6.0 ± 14.2	−25.7–31.7

SA: sulfonylurea; SD: standard deviation.

Cont: continuation of therapy; discount: 2 weeks of therapy discontinuation.

^#^visit 1, 2, and 3 and averaged (see materials and methods).

*P = 0.001 versus healthy volunteers.

**P < 0.05 versus healthy volunteers.

***P < 0.05 versus diabetic patients on metformin only.

## Abbreviations

ACE: Angiotensin-converting enzyme
AI: Augmentation index
BMI: Body mass index
BP: Blood pressure
CI: Confidence interval
CV: Coefficient of variation
FMD: Flow-mediated dilation
GFR: Glomerular filtration rate
HR: Heart rate
LUMC: Leiden University Medical Center
NO: Nitric oxide
PAT: Peripheral arterial tone
RHI: Reactive hyperemia index.
